# On the effect of walking surface stiffness on inter-limb coordination in human walking: toward bilaterally informed robotic gait rehabilitation

**DOI:** 10.1186/s12984-016-0140-y

**Published:** 2016-03-22

**Authors:** Jeffrey Skidmore, Panagiotis Artemiadis

**Affiliations:** Department of Mechanical and Aerospace Engineering, Arizona State University, 501 E. Tyler Mall, Tempe, USA

**Keywords:** Inter-leg coordination, Gait rehabilitation, Treadmill therapy

## Abstract

**Background:**

Robotic devices have been utilized in gait rehabilitation but have only produced moderate results when compared to conventional physiotherapy. Because bipedal walking requires neural coupling and dynamic interactions between the legs, a fundamental understanding of the sensorimotor mechanisms of inter-leg coordination during walking, which are not well understood but are systematically explored in this study, is needed to inform robotic interventions in gait therapy.

**Methods:**

In this study we investigate mechanisms of inter-leg coordination by utilizing novel sensory perturbations created by real-time control of floor stiffness on a split-belt treadmill. We systematically alter the unilateral magnitude of the walking surface stiffness and the timing of these perturbations within the stance phase of the gait cycle, along with the level of body-weight support, while recording the kinematic and muscular response of the uperturbed leg. This provides new insight into the role of walking surface stiffness in inter-leg coordination during human walking. Both paired and unpaired unadjusted t-tests at the 95 % confidence level are used in the approriate scernario to determine statistical significance of the results.

**Results:**

We present results of increased hip, knee, and ankle flexion, as well as increased tibialis anterior and soleus activation, in the unperturbed leg of healthy subjects that is repeatable and scalable with walking surface stiffness. The observed response was not impacted by the level of body-weight support provided, which suggests that walking surface stiffness is a unique stimulus in gait. In addition, we show that the activation of the tibialis anterior and soleus muscles is altered by the timing of the perturbations within the gait cycle.

**Conclusions:**

This paper characterizes the contralateral leg’s response to ipsilateral manipulations of the walking surface and establishes the importance of walking surface stiffness in inter-leg coordination during human walking.

## Background

Robot-assisted gait therapy has been explored as an alternative to conventional physiotherapy because robots can perform many repetitions with high accuracy [1]. Repetition is an important factor in facilitating neural plasticity [2] which is the basic mechanism underlying improvement in functional outcome after stroke [3, 4]. A variety of robotic devices have been proposed for gait rehabilitation [5–10] but have not produced superior results compared to conventional physiotherapy [11–14], which suggests that current robotic interventions are failing to stimulate the correct mechanism underlying the gait impairment.

A limitation of the robotic devices is that they do not consider mechanisms of inter-leg coordination and how the sensory feedback from one leg affects the motion of the other leg. Instead, the state of the art devices impose motion on the impaired limb. Human walking, in addition to running and stair climbing, requires inter-limb coordination and neural coupling [15]. A recent review suggests that utilizing inter-limb coupling in stroke rehabilitation therapies will lead to improved functional outcome [15]. As an example, chronic stroke patients performing seated bilateral leg exercises had increased step length during treadmill walking [16]. Therefore, a fundamental understanding of underlying sensorimotor mechanisms of inter-leg coordination may facilitate improved robotic interventions in gait therapy.

Investigation of the role of afferent sensory feedback to gait control mechanisms of inter-leg coordination usually involve sensory perturbations and the analysis of their effects. Various platforms and protocols have been used to investigate bilateral reflex mechanisms during different phases of the gait cycle [17–21], with the majority of the experimental protocols focusing on over-ground walking and dropping of the supportive surfaces at distinct gait phases [17, 18, 20, 21]. Perturbations to the load (i.e. force felt by the foot) feedback as well as the length of specific muscles during walking have been associated with evoked muscular activations of the unperturbed leg [19, 22–26]. For example, unloading of the plantarflexor muscles by unilaterally dropping the walking surface during stance phase significantly decreases soleus muscle activity of the contralateral leg [22].

One significant limitation of the previous studies is that the sensory perturbations presented in the previous experiments were almost exclusively caused by dropping the walking surface, which causes a disruption in both force and kinesthetic kinematic feedback. When the walking surface is dropped there is a change in leg kinematics, and the force feedback on the bottom of the foot is lost as the foot loses contact with the walking surface. These types of perturbations do not provide any separation of those two sources of sensory feedback, and do not allow further in-depth investigation of the role of force and kinesthetic feedback in gait. In order to answer important questions on inter-leg coordination and sensorimotor control, it is desirable, therefore, to differentiate force and kinesthetic feedback. Adjustment of the stiffness of the walking surface is a unique way to achieve this differentiation, since stepping on a low stiffness platform continues to provide force feedback but affects kinematics.

A few studies have utilized compliant surfaces in researching sensorimotor mechanisms in human locomotion including while stepping on/off, hopping, or walking on a compliant surface [27–32]. While the majority of these studies focus primarily on the perturbed leg or the center of mass of the walker [27, 29–31], the bilateral response has also been investigated [28, 32]. For example, the contralateral tibialis anterior was activated 140 ms later than the normal condition when healthy subjects unexpectedly stepped onto a foam mat in the walkway [32]. Another study has also shown that walking on a compliant surface creates activation of the tibialis anterior and soleus in both legs when compared to walking on a rigid surface [28]. However, these studies lack the ability to vary the magnitude and timing of the walking surface stiffness perturbations within the gait cycle.

Moreover, another limitation of all of the previously mentioned works, including those utilizing compliant surfaces, is that the previous studies have failed to separate the mechanisms of inter-leg coordination from that of balance support. As a result, mechanical perturbations and sudden load changes would have likely triggered reflex mechanisms and vestibular responses to maintan balance and stability. However, little is known whether the bilateral activations are exclusively caused by the mechanisms required for body stabilization and balance maintenance, or if it is also brought about from inter-limb coordination and mechanisms of gait. This lack of knowledge leaves a significant gap in our understanding of sensorimotor control of gait and the effect of surface stiffness on gait mechanisms.

In this paper we utilize the unique capabilities of the Variable Stiffness Treadmill (VST) system [33, 34] to investigate the role of surface stiffness in inter-leg coordination mechanisms by designing and applying unilateral low stiffness perturbations that evoke contralateral leg responses. The VST was chosen for this work because it has a wide range of controllable stiffness and the ability to apply stiffness perturbations in any phase of the gait cycle which allows for a more thorough investigation of the effect of surface stiffness on mechanisms of inter-leg coordination.

## Methods

### Experimental setup

**Fig. 1:**
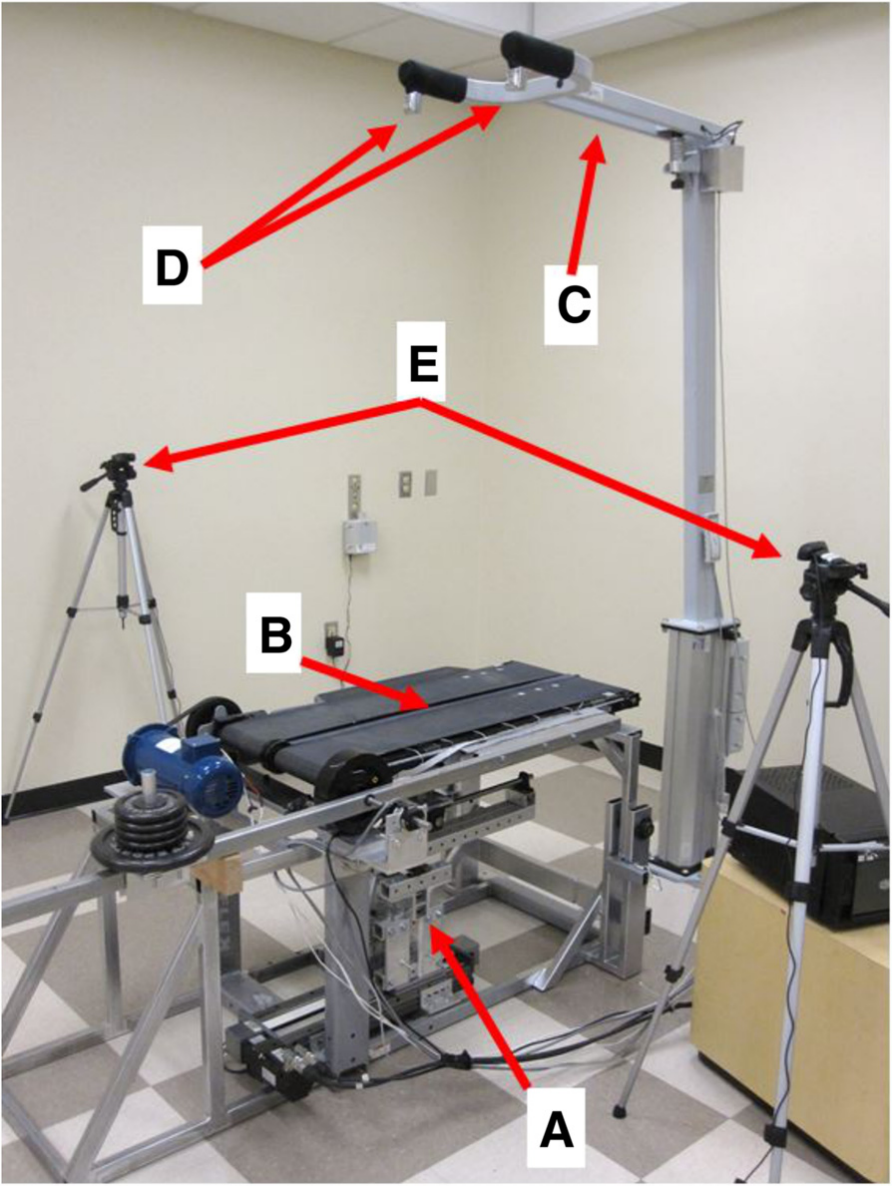
The Variable Stiffness Treadmill (VST) setup. Subsystems shown include: **a** Variable stiffness mechanism, **b** Split-belt treadmill, **c** Custom-made harness-based body-weight support, **d** BWS Loadcells, **e** Motion capture system

In order to understand the role of surface stiffness in inter-leg coordination during human walking, a variety of unilateral stiffness perturbations were induced using the Variable Stiffness Treadmill (VST) system [33, 34] shown in Fig. 1. The VST provides a unique platform for investigation of the role of walking surface stiffness in inter-leg coordination mechanisms. Advantages of the VST over other experimental platforms include (1) a wide range of controllable stiffness while maintaining high resolution, (2) the ability to apply low stiffness perturbations at any phase of the gait cycle and (3) body-weight support for the walker in order to suppress mechanisms of balance and posture. These advantages are created by combining a variety of components into one unique system. The major components of the VST include a variable stiffness mechanism, a split-belt treadmill, a custom-built body-weight support and a motion capture system. Each component will be discussed below.

#### Variable stiffness mechanism

**Fig. 2:**
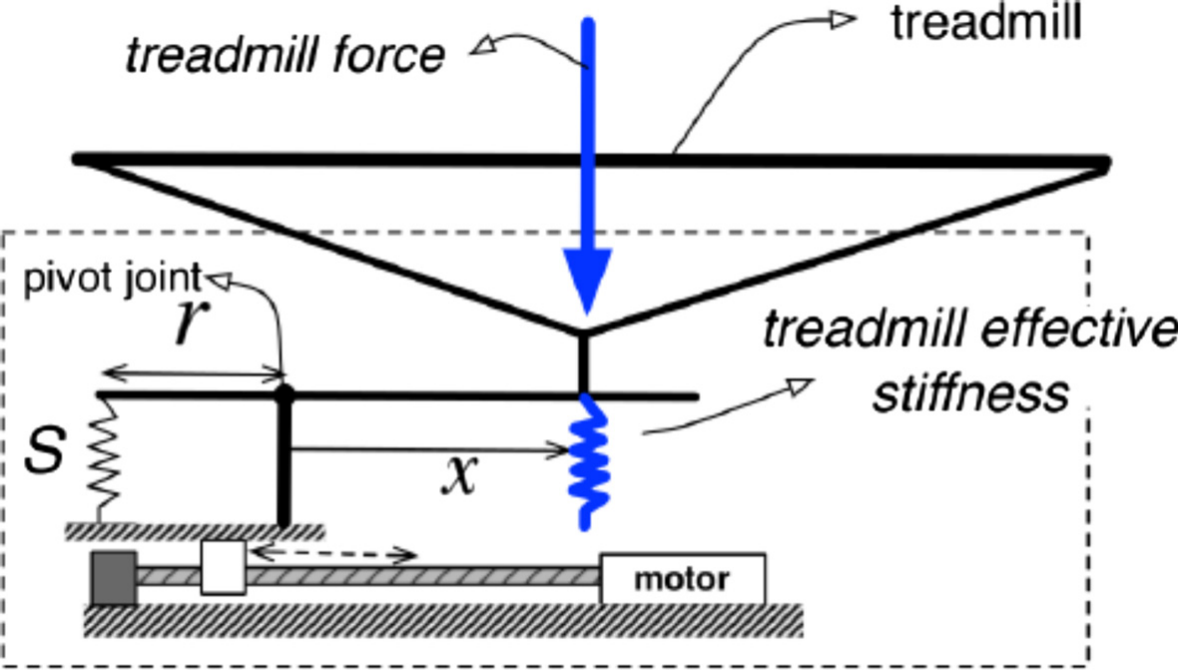
Conceptual diagram of the variable stiffness mechanism. The conceptual idea of a spring-loaded lever system mounted on a translational track behind the development of the variable stiffness mechanism

In its most simplified form, the variable stiffness mechanism is a spring-loaded lever mounted on a translational track, as shown in Fig. 2. The effective stiffness of the treadmill, located at a distance *x* from the pivot joint, is dependent on the coefficient of stiffness *S* of the linear spring and the moment arm through which it exerts a force [35]. By design, *S* and *r* remain constant, therefore, the effective stiffness of the treadmill can be controlled by changing the distance *x*. In the VST system, the distance *x* is controlled by placing the VST mechanism assembly onto the carriage of a high-capacity linear track (Thomson Linear, Part Number: 2RE16-150537) which is controlled by a high-precision drive (Kollmorgen, Part Number: AKD-P00606-NAEC-0000). The resolution of achievable displacement of the linear track is 0.01 mm. The device can change the surface stiffness from infinite (non-compliant walking surface) to 61.7 N/m (extremely low stiffness) in 0.13 s. It can also reach any stiffness in between at a maximum resolution of 0.038 N/m. Therefore, the VST can create quick, high resolution stiffness perturbations of nearly any magnitude during any phase of the gait cycle. This leads to consistent, repeatable, and unanticipated stiffness perturbations that are useful for altering kinematic feedback. The vertical stiffness of the walking surface is calculated from the knowledge of the subject’s foot position, the force exerted by the subject on the treadmill, and the angular deflection of the treadmill. For a detailed characterization of the variable stiffness mechanism and a complete analysis of the VST see [34].

#### Split-belt treadmill

The VST employs a split-belt treadmill configuration in order to allow each belt to deflect independently. The treadmill belts are supported at 70 cm above the floor on a frame of steel tubing that permits each belt to independently deflect downward to a maximum of 30° from the horizontal position. This will allow one leg to experience low stiffness perturbations while the other leg remains supported by a rigid surface. The split-belt treadmill is shown in Fig. 1, part B.

#### Body weight support

Separate from the treadmill structure there is a custom-built body-weight support designed by LiteGait. By adjusting the height of the support system, full or partial body-weight support can be selected. This is an important capability to reduce activation of body stabilization and balance maintenance mechanisms. In addition, the support increases safety and extends the system’s capabilities to stroke patients and other individuals with decreased mobility and stability. Two loadcells attached on the body-weight support harnesses are measuring the subject’s weight supported by the mechanism from each side. The body-weight support and loadcells are shown in Fig. 1, parts C and D, respectively.

#### Motion capture

Another important component of the VST is a low-cost and portable motion capture system comprised of infrared cameras (Code Laboratories Inc, model: DUO MINI LX) and infrared LEDs (Super Bright LEDs Inc, model: IR-1WS-850). The motion capture is important for tracking the location of the subject’s foot in order to maintain the desired stiffness underneath the walker, and for precise timing of stiffness perturbations within the gait cycle. The motion capture system is also used for recording lower-limb joint angles throughout the gait cycle. The two cameras tracking the two legs are shown in Fig. 1, part E.

### Experimental protocol

In order to investigate the role of walking surface stiffness in inter-leg coordination, we investigated the response of the contralateral (unperturbed) leg to unilateral stiffness perturbations while varying three different experimental parameters. The variables that were changed in three separate experiments were (1) the magnitude of the stiffness perturbation, (2) the level of supplied BWS and (3) the timing of the stiffness perturbation within the gait cycle. In each experiment the subject walked on the treadmill at a speed of 0.60 m/s and the differentiating aspects of the protocol for each experiment will be discussed below. While results from the experiment involving the change of stiffness magnitude (experiment 1) have been presented previously by the authors [36, 37], they are included in this work for completeness. The investigation of the effect of surface stiffness on mechanisms of inter-leg coordination through the systematic manipulation of experimental conditions (as presented in this work) would not be complete without presenting the effect of the change in magnitude of the stiffness perturbations. For each experiment, five healthy subjects with no known neurological or gait impairments participated, where the five subjects were different for each experiment. Informed consent from the subject was obtained at the time of each experiment, and each experimental protocol is approved by the Arizona State University Institutional Review Board (IRB ID#: STUDY00001001).

#### Experiment 1: Altered stiffness magnitude

**Fig. 3:**
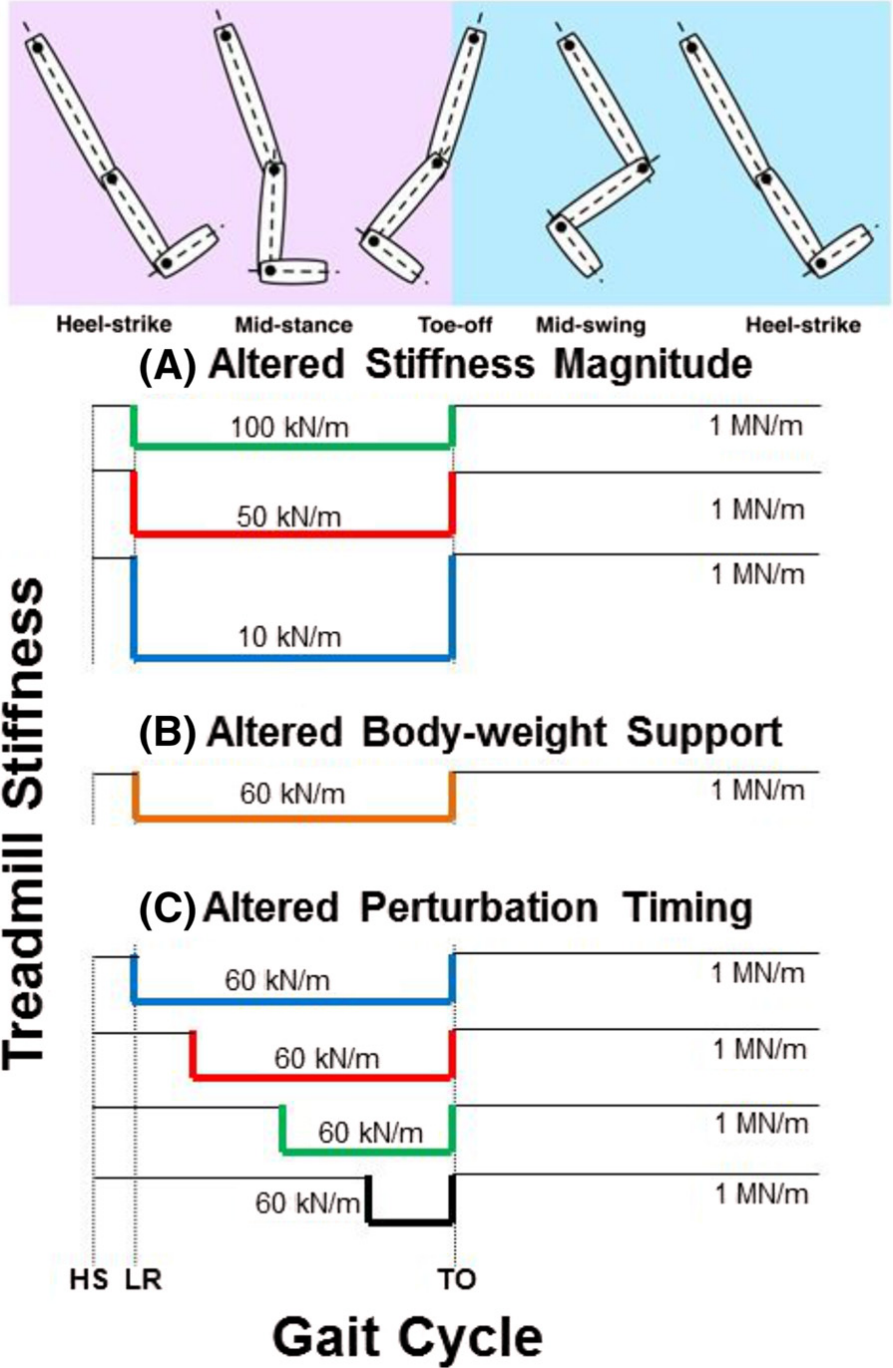
Timing and magnitude of unilateral stiffness perturbations. A diagram indicating the timing and magnitude of unilateral stiffness perturbations for the three experiments presented in this work: **a** Altered Stiffness Magnitude, **b** Altered BWS and **c** Altered Perturbation Timing. Heel strike, loading response and toe-off are represented by HS, LR and TO, respectively

For this experiment, five healthy subjects [age 25 ± 5.4 years, weight 845 ± 156 *N*] walked on the treadmill for at least 200 gait cycles while being supported with approximately 30 % BWS. A value of 30 % BWS was chosen because this level of support has been given in other studies [38, 39] and effectively provides balance support without eliminating somatosensory feedback by unloading too much of the subject’s weight. The right treadmill belt was not allowed to deflect for the duration of the experiment thus preventing any direct perturbation of the right leg. The surface underneath the left leg was commanded to maintain a stiffness of 1000 kN/m, which makes the treadmill very stiff (i.e. considered to be rigid), for 30 gait cycles at the beginning of the experiment. Then, after a random number *n* of steps, where *n*∈[3,7], the stiffness was immediately dropped to 1 of 3 values: 10, 50 or 100 kN/m. The low stiffness perturbation began approx. 130 ms after heel-strike and lasted for the duration of the left leg stance phase (i.e. until toe-off) after which the stiffness was commanded back to 1000 kN/m for the next *n* number of steps. A graphical representation of the timing and magnitude of the stiffness perturbations is shown in Fig. 3(a). An average of 17 ± 2.3 perturbations at each stiffness level were experienced by all subjects.

#### Experiment 2: Altered BWS

This experiment was broken up into four sub-experiments, where the only difference between each sub-experiment was the level of BWS (0, 10, 20 or 30 %) provided to the subject. Five healthy subjects [age 24 ± 2.4 years, weight 714 ± 108 *N*] walked on the treadmill for at least 100 gait cycles while being supported with the selected level of BWS. Similar to the previous experiment, the surface underneath the left leg was commanded to maintain a stiffness of 1000 kN/m and then, after a random number *n* of steps, where *n*∈[3,7], the stiffness was immediately dropped to 60 kN/m approx. 130 ms after heel-strike. The perturbation lasted for the duration of the left leg stance phase after which the stiffness was commanded back to 1000 kN/m for the next *n* number of steps. A walking surface stiffness of 60 kN/m was used for each level of BWS and was chosen because it is an intermediate value in the range of stiffness perturbations used in the first experiment. A graphical representation of the timing and magnitude of the stiffness perturbation for this experiment is shown in Fig. 3(b). All subjects experienced 15 perturbations of the walking surface stiffness at each level of BWS. Again, the right treadmill belt was not allowed to deflect for the duration of the experiment thus preventing any direct perturbation of the right leg kinematics.

#### Experiment 3: Altered perturbation timing

This experiment was similar to the first experiment, except that instead of changing the magnitude of the stiffness perturbation, the timing of a stiffness perturbation of constant magnitude (60 kN/m) was altered. In this experiment, five healthy subjects [age 25 ± 3.6 years, weight 756 ± 165 *N*] walked on the treadmill for at least 150 gait cycles while being supported with approximately 30 % BWS. A value of 30 % BWS was chosen to provide some balance support and to allow for comparison with the first experiment. The surface underneath the left leg was commanded to maintain a stiffness of 1000 kN/m for 30 gait cycles at the beginning of the experiment. Then, after a random number *n* of steps, where *n*∈[5,7], the stiffness immediately dropped to a level of 60 kN/m when the middle of the subject’s left foot reached a certain percentage of the left stance phase. A stiffness magnitude of 60 kN/m was chosen in order to be consistent with the second experiment. The perturbation began at one of four locations (12, 30, 55 or 80 % of the stance phase) that was randomly selected and lasted until the end of the left leg stance phase after which the stiffness was commanded back to 1000 kN/m for the next *n* number of steps. A graphical representation of the timing and magnitude of the stiffness perturbations for this experiment is shown in Fig. 3(c). An average of 9 ± 2.8 perturbations at each of the four timing instances were experienced by all subjects. Again, the right treadmill belt was not allowed to deflect for the duration of the experiment thus preventing any direct perturbation of the right leg.

### Data analysis

The data analysis of the kinematic and muscular response of the unperturbed leg was the same for each of the three experiments described above. In all of the experiments, kinematic data for both legs were obtained at 140 Hz using the previously mentioned infrared camera system that tracked 12 (6 on each leg) infrared LEDs placed as pairs on the thigh, shank, and foot. The muscle activity of the unperturbed leg was obtained using surface electromyography (EMG) via a wireless surface EMG system (Delsys, Trigno Wireless EMG) and recorded at 2000 Hz. Electrodes were placed on the tibialis anterior (TA) and soleus (SOL) of the right leg. Raw EMG signals were processed by finding the moving root mean square envelope of each signal with a 250 ms window. After computing the EMG linear envelope, the data were normalized to the maximum value of that EMG signal. EMG electrodes were not placed on the left leg because the focus of this work is to understand inter-leg coordination in human walking by investigating the response of the unperturbed leg to unilateral stiffness perturbations. Therefore, even though the left leg was directly perturbed through the stiffness change of the left walking surface, the analyses for the rest of the paper will be focused on the effects of the perturbation on the response of the contralateral leg. Moreover, we have shown in previous studies that the ipsilateral leg kinematics are significantly affected by stiffness perturbations [33, 34]. Thus, presenting results of the perturbed leg is redundant and not within the scope of the present study and will, therefore, not be presented in this work.

The kinematic and EMG data corresponding to the gait cycles of normal conditions and the cycles pertaining to the perturbations were found and normalized temporally to percent gait cycle in order to eliminate discrepancies due to natural variations in gait patterns (i.e. stride length, cycle duration, etc). The data of each gait cycle was resampled at each 0.1 % of the gait cycle (approximately 1.5 ms). The first 30 gait cycles and the cycles in between perturbations during the normal conditions are included in the unperturbed data set. One or two cycles (depending on which of the three experiments) following a perturbation are not included in the unperturbed set in order to eliminate any residual effects from the perturbation. This results in normalized kinematic and EMG signals as a function of percent gait cycle, where 0 and 100 % correspond to the heel-strike of the left leg.

Two different t-tests were utilized to establish statistical significance of the results. In order to evaluate the significance of recorded responses in both kinematics and EMG when compared to the normal condiction, statistical significance was determined using an unadjusted *unpaired* t-test at each time instance. The unpaired t-test was selected in this case because it is a comparison of two independent distributions (i.e. gait cycles with and without perturbation) which have similar variances but different sample sizes. In order to evaluate the significance of perturbations across subjects, the *paired* t-test was used. Two values (the mean amplitude of cycles with and without perturbations) from each subject were used to test the significance of response to the perturbation at each time instance. Each test was performed at the 95 % confidence level and any potential Type I errors from tests being performed at each 0.1 % of the gait cycle were eliminated by only concluding significance if at least 40 tests (i.e. 4 % of the gait cycle) in a row indicated significance.

A latency of response in each experiment is calculated from the beginning of the perturbation until there is a statistically significant difference between the TA EMG magnitude recorded during the perturbation and normal conditions.

## Results

### Experiment 1: Altered stiffness magnitude

**Fig. 4:**
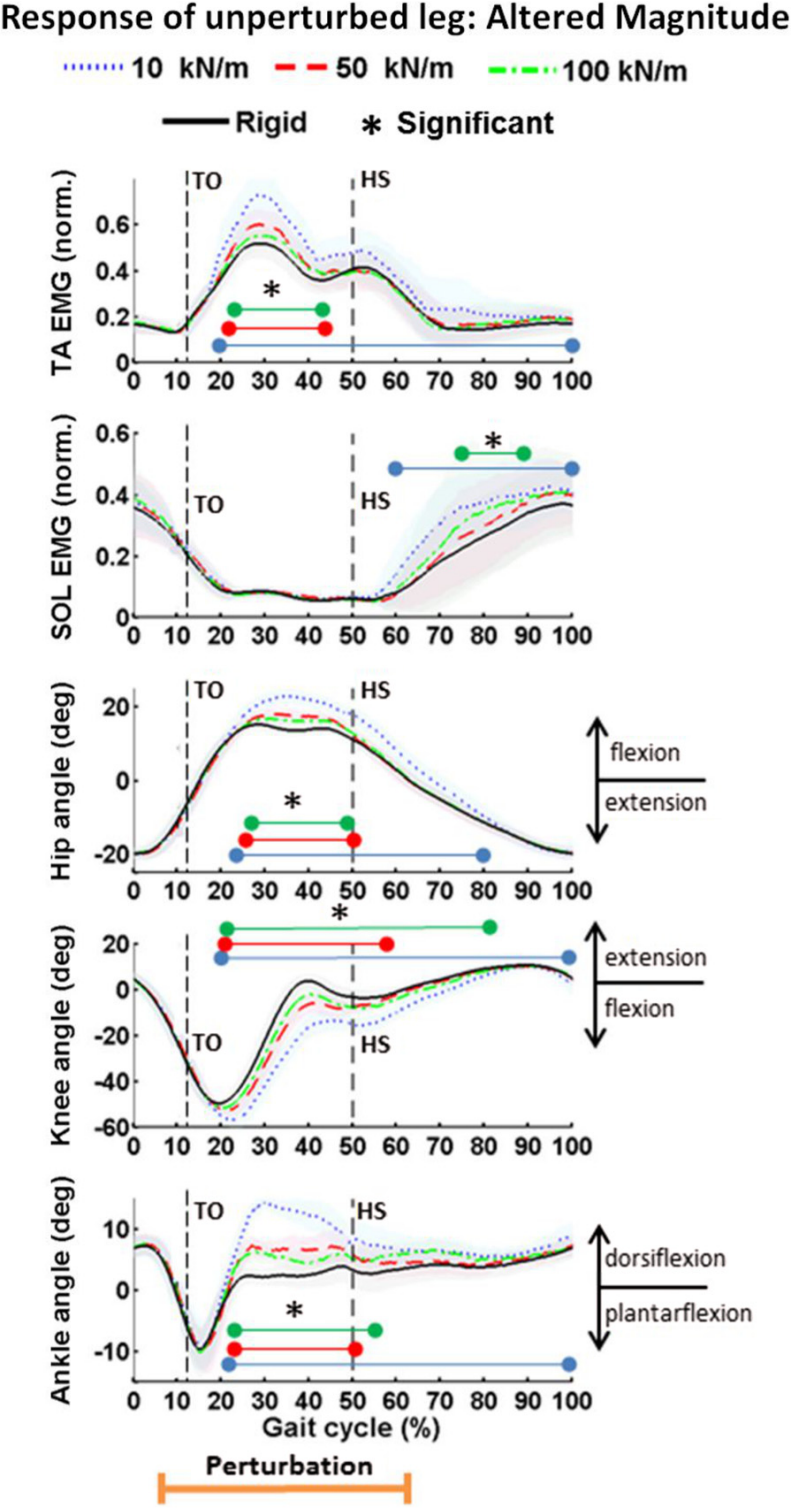
Response of unperturbed leg: Altered Magnitude. Averaged muscle activity and joint kinematics of the unperturbed (right) leg for a representative subject. Plotted from top to bottom is the normalized TA EMG, normalized SOL EMG, hip flexion (+) - extension (-), knee flexion (-) - extension (+) and ankle dorsi (+) - plantar (-) flexion for gait cycles at each of four surface stiffness levels. Mean (darker lines) and standard deviations (lightly shaded areas) values are shown along with an indication of the timing of the perturbation. Statistically significant changes are indicated by colored bars (corresponding to each stiffness level, aligned vertically from highest to lowest stiffness) that are placed beneath a black asterisk. Heel-strike and toe-off of the right leg are indicated by HS and TO, respectively. The duration of the gait cycles shown is approximately 1.8 s

As mentioned previously, the results for this experiment have been presented before [36, 37], but are included in this work for completeness of analysis. The kinematic and muscular response to unilateral low stiffness perturbations of the walking surface of different magnitudes for a representative subject is shown in Fig. 4. Only data for a representative subject is shown in Fig. 4 for clarify of presentation, but the contralateral response was consistent across subjects.

The experimental data of the response of the unperturbed leg shows a systematic evoked response in both kinematics and muscular activity. The majority of these evoked changes begin near 22 % and then converge back to the normal walking pattern later in the gait cycle. The evoked response was statistically significant at the 95 % confidence level for a two sample unpaired t-test when comparing the data for each perturbation level to the rigid (i.e. normal) condition. Colored bars indicating when significant changes are observed are included in Fig. 4. As can be seen, the significance of the response is dependent on the magnitude of the stiffness perturbation where lower stiffness values result in greater contralateral response.

Moreover, the response in the contralateral TA is scalable such that as the magnitude of the perturbation increases (i.e. lower stiffness values), there are increased changes in TA activation. The significant increase in TA activation with lower walking surface stiffness is shown by using a paired t-test to compare the mean TA values at two levels of stiffness for all subjects. For example, the evoked TA EMG during the lowest stiffness perturbations (10 kN/m) is significantly greater than the medium stiffness perturbations (50 kN/m) from 16.8 to 42.5 percent of the gait cycle. This is shown in the first row of Table 1. The time period (in percent gait cycle) when the evoked TA EMG is significant when comparing the other levels of stiffness is also shown in Table 1. As calculated from the last column in the table, the TA activation significantly increases at each level of stiffness for at least 13 % of the gait cycle. Therefore, there is a scalable response of the contralateral TA in response to unilateral stiffness perturbations. While the same systematic and scalable evoked response is not apparent in the SOL, there is significant evoked activity in the SOL during stance phase of the right leg.

**Table 1:** Timing of evoked tibialis anterior activation for experiment 1

Stiffness level 1	Stiffness level 2	Range of significance
(kN/m)	(kN/m)	(% gait cycle)
10	50	16.8 - 42.5
50	100	25.3 - 38.8
100	1000	21.0 - 36.0

This table contains the range in percent gait cycle when the statistically significant evoked tibialis anterior EMG is seen when comparing two levels of stiffness

### Experiment 2: Altered BWS

**Fig. 5:**
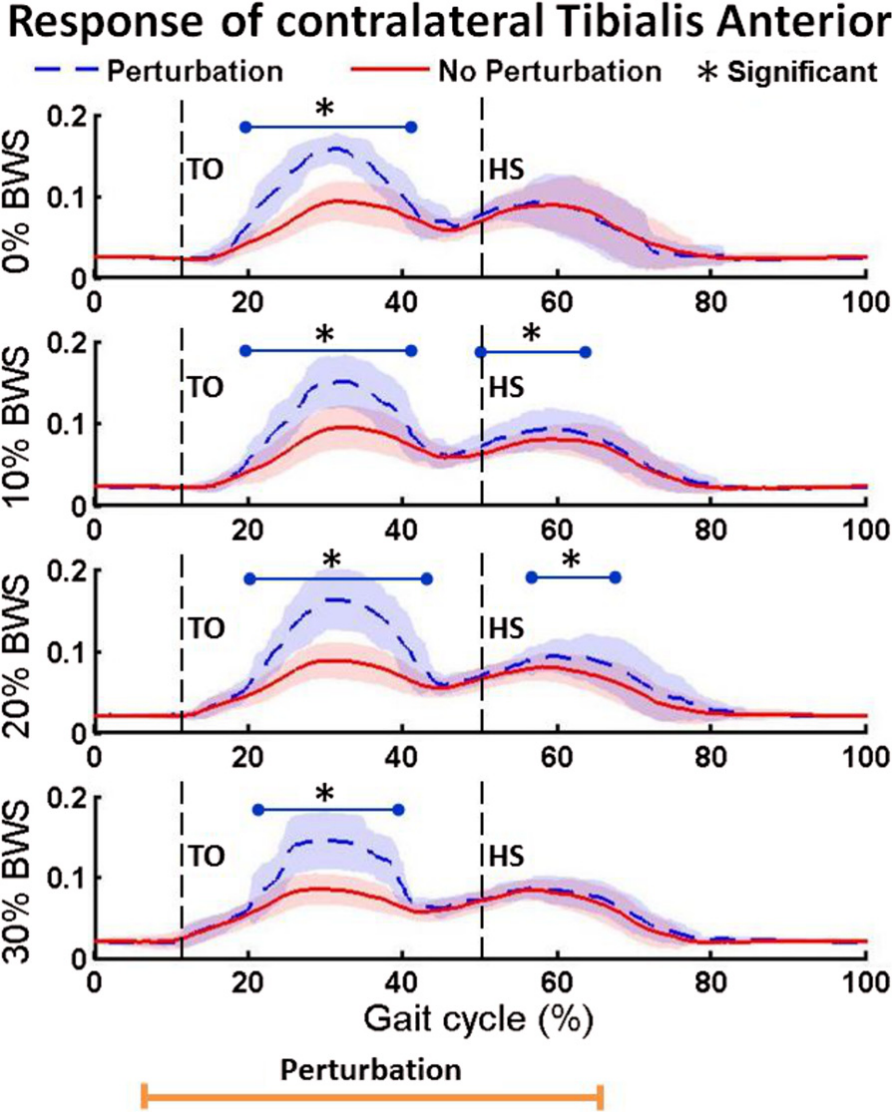
Response of contralateral Tibialis Anterior. Averaged TA muscle activity for perturbed and unperturbed gait cycles at each of the four levels of BWS for a representative subject. Mean (*darker lines*) and standard deviation (*lightly shaded areas*) values are shown along with an indication of the timing of the perturbation. Statistically significant changes in perturbed gait cycles are indicated by a blue bar and a black asterisk. Heel-strike and toe-off of the right leg are indicated by HS and TO, respectively

We first present the evoked muscle activity of the TA in the perturbed leg for all levels of BWS for comparison and validation with the first experiment. The normalized EMG amplitude for the TA (mean and standard deviation) for all perturbed and unperturbed gait cycles pertaining to each level of BWS for a representative subject is shown in Fig. 5.

**Fig. 6:**
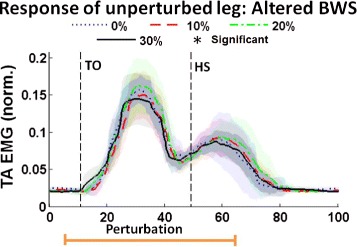
Response of unperturbed leg: Altered BWS. Averaged TA muscle activity for gait cycles at each of the four levels of BWS for a representative subject. Mean (*darker lines*) and standard deviation (*lightly shaded areas*) values are shown along with an indication of the timing of the perturbation. The timing of toe-off and heel-strike of the right leg within the gait cycle are represented by TO and HS, respectively

Similar to experiment 1, there is a statistically significant increase in TA activity between approx. 20 to 40 % of the gait cycle, as well as occasionally at other times later in the gait cycle (such as shortly after heel-strike of the right leg). The significant evoked muscle activity during swing phase is observed for all levels of BWS and is consistent across subjects. The time (in percent gait cycle) of when significant TA EMG is first seen (mean and standard deviation across all levels of BWS) for all subjects is shown in Table 2. While there is some variability in the onset of evoked activity, all subjects show evoked TA activity during the swing phase of the gait cycle and at all levels of BWS. cv Of significant importance is that there is no statistically different response between the evoked TA activity at each level of BWS. The TA activation (mean and standard deviation) during perturbation cycles for all levels of BWS (i.e. all of the blue lines from Fig. 5) is shown in Fig. 6. As can be seen, the responses are very similar and there is no statistically significant difference at any time during the gait cycle. Therefore, the level of evoked muscle activity for a constant stiffness perturbation of 60 kN/m is independent of the level of BWS.

**Table 2:** Timing of evoked tibialis anterior activation for experiment 2

Subjects				
1	2	3	4	5
26 *%*±1.7	19 *%*±0.8	21 *%*±2.0	20 *%*±5.0	28 *%*±1.6

This table contains the mean and standard deviation in percent gait cycle across all levels of body weight support for when the statistically significant evoked tibialis anterior EMG is first seen for all subjects

### Experiment 3: Altered perturbation timing

**Fig. 7:**
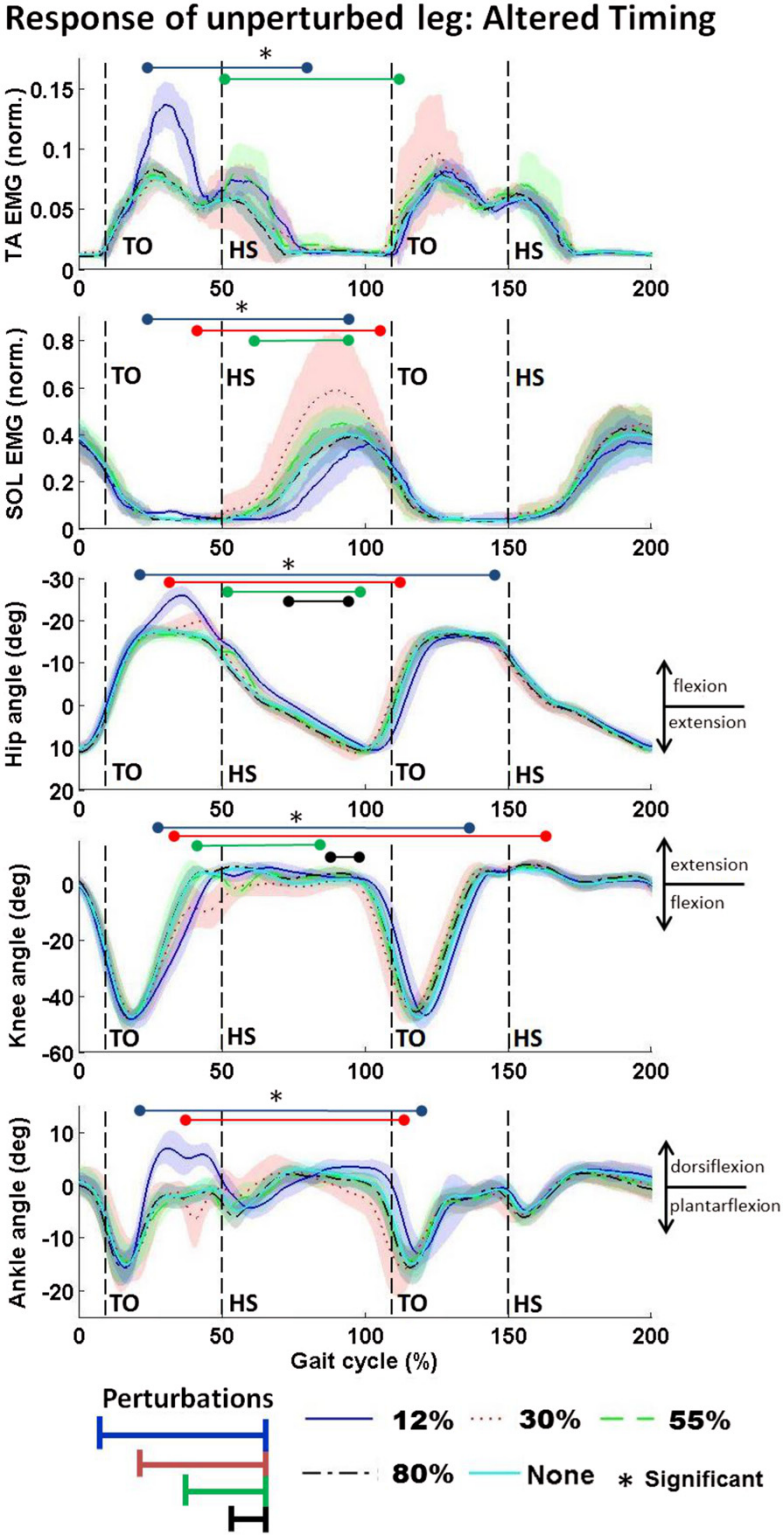
Response of unperturbed leg: Altered Timing. Averaged muscle activity and joint kinematics of the unperturbed (right) leg for a representative subject. Plotted from top to bottom is the normalized TA EMG, normalized SOL EMG, hip flexion (+) - extension (−), knee flexion (−) - extension (+) and ankle dorsi (+) - plantar (−) flexion. Mean (darker lines) and standard deviations (lightly shaded areas) values for gait cycles pertaining each timing of the perturbation are shown along with an indication of the timing of each perturbation. Statistically significant changes are indicated by colored bars (corresponding to each perturbation timing, aligned vertically from earliest to latest in the gait cycle) that are placed beneath a black asterisk. Heel-strike and toe-off of the right leg are indicated by HS and TO, respectively. The duration of the data plotted is approximately 3.3 s

The kinematic and muscular response due to variation in the onset of the low stiffness perturbations for a representative subject is shown in Fig. 7. The results from this experiment indicate that the timing of the low stiffness perturbation affects the timing of the muscular and kinematic response of the unperturbed leg. As would be expected, the data show that the altered response is only seen after the perturbation, independent of the onset of the perturbation. Of importance to note is that statistically significant evoked muscle activity is primarily seen when the muscle is normally active, independent of the timing of the perturbation. As seen in Figs. 4, 5 and 7, the majority of the evoked TA and SOL activity is seen during the swing and stance phases, respectively, which is when these muscles have higher activity during human walking. The timing of the evoked response in both the TA and SOL is consistent across subjects. The time (in percent gait cycle) of when significant TA and SOL EMG begin (mean and standard deviation across all subjects) for all timing instances of the perturbation is shown in Table 3. Only the beginning and not the end is shown because the durations of the significance varied between subjects. The important result here is that the onset of evoked contralateral response is consistent across subjects independent of when the timing of the perturbation begins.

**Table 3:** Timing of statistically significant changes for experiment 3

Muscle	Timing of perturbation (% stance phase)			
	12 %	30 %	55 %	80 %
Tibialis Anterior	23 *%*±3.6	42 *%*±1.4	56 *%*±5.7	-
Soleus	34 *%*±14	50 *%*±23	58 *%*±4.6	-

This table contains the mean and standard deviation in percent gait cycle across all subjects for when statistically significant response is first seen for the TA and SOL at all timing instances of the perturbations. No significant evoked response was seen with perturbations beginning at 80 % of the stance phase which is indicated with a dash

## Discussion

The results presented in this paper indicate that walking surface stiffness is a significant stimulus in gait. Moreover, the timing of evoked muscle activity in the contralateral leg suggests that the feedback may be modulated by supra-spinal neural circuits. Discussion of the importance of walking surface stiffness in gait and the potential application in robot-assisted gait therapy will be presented below.

### Stiffness stimulus

As mentioned previously, stiffness control provides a unique way to perturb leg kinematics while allowing the foot to maintain contact with the walking surface. The repeatability (consistency across subjects and experiments) and scalability (significant increase in EMG activity with decreasing stiffness) suggest that the stiffness of the walking surface is an important stimulus in gait.

Moreover, the consistent latency of evoked muscle activity after the perturbation in the results suggest that supra-spinal neural circuitry is stimulated through sudden low stiffness perturbations. The latency averaged across subjects and experiments resulted in a mean of 202 ± 60 ms. A delay of this duration corresponds to transcortical circuitry [40] suggesting that supra-spinal regions are stimulated through the low stiffness perturbations used in this study. Electroencephalography (EEG) recordings during recent experiments have revelaved that significant changes in brain activity within the sensorimotor region are evoked by the stiffness perturbations [41].

In addition, the timing of the evoked EMG in both the TA and SOL within the gait cycle suggests that supraspinal structures modify the amplitude of the neuromuscular response to sensory stimuli created by sudden changes in surface stiffness but does not initiate activation of the muscles in gait. This is consistent with the theory that supraspinal structures are not responsible for generating basic gait motor patterns through cyclical flexion and extension of the joints, but rather in modulating these basic gait patterns with descending inputs [42]. As can be seen in Figs. 4, 5, and 7, the majority of the evoked muscle activity occurs only when the muscle is normally active. Specifically, evoked EMG in the TA is seen during the swing phase and beginning of the stance phase, with the greatest change in EMG occurring at the same time (approx. 30 % gait cycle) as the peak EMG during normal walking. The same pattern is seen for the SOL. Even though the perturbation occurs from approx. 8 to 60 % of the gait cycle, evoked muscle activity of the SOL is not seen until the stance phase later in the gait cycle, which is when the SOL is active in normal walking.

In addition to all of the above, the experiment performed with different levels of BWS (experiment 2) suggests that stiffness is an important stimulus because the low stiffness perturbations evoke the same amplitude of contralateral TA activity despite changes in load force created by changes in BWS, as was shown in Figs. 5 and 6. Since the evoked muscle activity is independent of the level of supplied BWS, we conclude that walking surface stiffness, independent of force feedback, is important in gait.

### Possible medical application

There is a need to develop and investigate therapeutic protocols that utilize inter-limb coordination [15], and this study provides insight into the potential role of surface stiffness in robotic gait therapy. We show that muscular activity in the contralateral leg can be evoked through supra-spinal neural circuits by altering the surface stiffness below the ipsilateral leg in healthy subjects. We show scalable and repeatable increases in TA activation with different magnitude of walking surface stiffness. This result suggests a possible novel approach to robot-assisted gait therapy for hemi-paretic stroke patients of manipulating the healthy leg in order to provide therapy to the impaired leg. Moreover, because of the predictability of the timing and amplitude of the evoked response, a controller could be designed and tuned to evoke the desired muscle activity in a therapy session. Also, considering that the level of BWS does not appear to alter the evoked response, this therapy could be applied to walkers of differing levels of impairment, requiring varying levels of BWS.

Moreover, the evoked TA activity during swing phase that is presented in this study is an exciting result from a clinical perspective. A main deficiency in stroke survivors is insufficient TA activity (which is the primary muscle creating dorsiflexion) in the swing phase which results in decreased dorsiflexion (toe-up motion). Insufficient dorsiflexion during walking, referred to as drop-foot, is a problem that many impaired walkers suffer from, and is the leading cause of after-stroke falls [43]. Since circuits of neural coupling exist in poststroke patients as they do in healthy subjects [15], the results presented in this study for healthy subjects provide indications of the feasibility of a solution to drop-foot by manipulating the non-paretic leg in stroke patients through stiffness perturbations. Therefore, walking surface stiffness may play a significant role in robotic gait rehabilitation.

## Conclusions

This paper presents results of evoking kinematic and muscular changes in the contralateral leg of healthy subjects using unilateral low stiffness perturbations of the walking surface. By systematically altering the magnitude and timing of the stiffness perturbations, along with the level of body-weight support, we provide new insight into the role of surface stiffness in inter-leg coordination during human walking. The presented study demonstrates the importance of the stiffness of the walking surface as a stimulus in human gait. This importance is indicated by systematic changes in contralateral tibialis anterior activation, as well as lower limb joint angles, in response to unilateral stiffness perturbations. In addition, a latency greater than 200 *ms* from the beginning of the perturbation to the evoked response that was observed during all experiments suggests the existence of supra-spinal mechanisms of inter-leg coordination. Moreover, the magnitude and latency of evoked muscle activity did not vary with different levels of body-weight support which suggests that walking surface stiffness is an important stimulus in gait independent of the level of body-weight support that is provided.

The results presented in this paper provide novel insight into the role of sensory feedback, specifically surface stiffness, in inter-leg coordination during human gait. Moreover, the repeatability and scalability of the evoked muscle activity presented in this paper suggest the feasibility of using walking surface stiffness perturbations in gait rehabilitation. Future work will include testing of this hypothesis with hemi-plegic stroke patients and other impaired walkers.
